# Yokukansankachimpihange Improves the Social Isolation-Induced Sleep Disruption and Allopregnanolone Reduction in Mice

**DOI:** 10.3389/fnut.2020.00008

**Published:** 2020-02-11

**Authors:** Kenta Murata, Feng Li, Kanako Shinguchi, Misaki Ogata, Nina Fujita, Ryuji Takahashi

**Affiliations:** Kampo Research Laboratories, Kracie Pharma, Ltd., Takaoka, Japan

**Keywords:** Yokukansankachimpihange, insomnia, social isolation stress, GABA, allopregnanolone

## Abstract

Yokukansankachimpihange (YKSCH), a traditional Japanese medicine composed of 9 crude drugs, is designed to improve neurosis, insomnia in adults, and night crying in children. YKSCH has been reported to improve diurnal rhythm in patients with Alzheimer's disease and prolong the total sleeping time in healthy subjects. However, little is known about how YKSCH alleviates sleep disorders. Here, we investigated whether and how YKSCH treatment affected sleep latency and duration in group-housed and socially isolated mice. Male ddy mice were treated with YKSCH [1,500 mg/kg, per os (p.o.)] in group-housed or socially isolated conditions for 3–4 weeks. After the last injection, mice were intraperitoneally (i.p.) administered with pentobarbital (60 mg/kg) and the sleep latency and duration was evaluated. The results show that pretreatment with YKSCH had no effect on sleep latency or duration in group-housed mice. However, YKSCH treatment significantly improved the reduced sleep duration in socially isolated mice. This effect of YKSCH was inhibited by the administration of bicuculline (3 mg/kg, i.p.), a GABA_A_ receptor antagonist. Furthermore, we showed that YKSCH treatment improved the decrease in allopregnanolone content and its synthase expression levels in the olfactory bulb. These results suggest that YKSCH treatment improved social isolation stress-induced insomnia via the GABAergic pathway and that the mechanism of action of YKSCH is partly due to improvement of allopregnanolone levels of expression.

## Introduction

Yokukansankachimpihange (YKSCH), a traditional Japanese medicine, is designed to improve neurosis, insomnia in adults, and night crying in children. YKSCH is composed of 9 herbs: Pinellia tuber, Atractylodes rhizome, Poria sclerotium, Cnidium rhizome, Citrus unshiu peel, Japanese Angelica root, Bupleurum root, Uncaria hook, and Glycyrrhiza. In addition to traditional use, YKSCH has been approved for administration to patients with dementia in Japan to treat behavioral and psychological symptoms of dementia (BPSD). A recent clinical trial revealed that YKSCH treatment improves the Neuropsychiatric Inventory scores of “agitation,” “delusion,” and “sleep and night-time behavior change” in patients with Alzheimer's disease (1). Other research revealed that a combination treatment of YKSCH with donepezil improved diurnal rhythm in patients with Alzheimer's disease (2). Furthermore, YKSCH treatment prolongs the total sleeping time and tends to increase sleep efficacy based on polysomnography recordings in normal young healthy subjects (3). YKSCH treatment has also been reported to reduce memory impairment and BPSD-like behavior, such as hallucinations and aggressive behavior, in several rodent models (4–7). Although some research using animal models have begun to elucidate the action of YKSCH in the central nervous system, little is known about how YKSCH alleviates insomnia observed in clinical practice.

Social isolation stress is reported to have negative effects on longevity as well as physical and mental conditions such as depression, fatigue, and sleep disruption (8, 9). Sleep disruption leads to a reduction in work productivity and personal quality of life, such as working deficits, day time sleepiness, depression, and attention and learning problems. In addition, a reduction of sleep quality is also reported to trigger a reduction in social interactions (10). On the other hand, social isolation stress in rodent models also leads to the same abnormal behaviors observed in humans. Mice housed in social isolation for 3–4 weeks develop increased anxiety-like and aggressive behavior and display decreased responsiveness to drugs that stimulate aminobutyric acid type A (GABAA) receptors such as pentobarbital (11, 12). Hence, socially isolated animal have been used as a model to evaluate the effect of sedative drugs. Changes in various neurotransmitter systems were reported in socially isolated stress animals, including dopaminergic (13), serotonergic (14), and noradrenergic systems (15, 16). Among these neurotransmitter systems, several reports showed that the reduction of responsiveness to pentobarbital in this model was related to the dysfunction of the GABAergic neurotransmitter system and the reduction of neurosteroid biosynthesis in the brain. Allopregnanolone (ALLO) is the cholesterol-derived neurosteroid in the brain; it declines with age and because of neurodegenerative diseases (17, 18). ALLO acts as a positive allosteric modulator of the GABA_A_ receptor, and ALLO content in the olfactory bulb (OB) and prefrontal cortex (PFC) is reduced by long-term social isolation (19).

The purpose of this study was to investigate whether and how YKSCH enhances pentobarbital-induced sleep in group-housed and socially isolated mice.

## Materials and Methods

### Animals

Six-week-old male Slc;ddY mice, weighing 32.1–35.5 g (mean ± SD = 33.6 ± 0.9 g), were purchased from SLC (Shizuoka, Japan). Animals were housed in sterilized polypropylene cages (4 mice/cage) and provided laboratory pellet chow (CE-2, Clea Japan Inc., Tokyo, Japan) and water *ad libitum* at 24 ± 2°C under a 12 h light–dark cycle (lights on from 8:00 to 20:00). Before experimental procedures, they were acclimatized to the room for 1 week. Behavioral experiments were performed between 9:00 and 18:00, except for the locomotor activity test. All efforts were made to minimize both the suffering of and the number of animals used. This study was carried out in accordance with the principles of the Basel Declaration and recommendations of guidelines for Proper Conduct of Animal Experiments, the Experimental Animal Care Committee of Kracie Pharma, Ltd. (Toyama, Japan). The protocol was approved by the Experimental Animal Care Committee of Kracie Pharma, Ltd.

### Plant Materials and Preparation of the Extract

YKSCH is composed of nine dried medical herbs, including Pinellia tuber, Atractylodes rhizome, Poria sclerotium, Cnidium rhizome, Citrus unshiu peel, Japanese Angelica root, Bupleurum root, Uncaria hook, and Glycyrrhiza (Table 1), and is supplied by Kracie Pharma, Ltd. as a formulation (EK-83). Each plant material was identified based on its external morphology and was authenticated by compound markers of plant specimens according to the method of the Japanese Pharmacopeia and our company's standard. EK-83 (lot No.06MH) was suspended in distilled water immediately before use and was administered orally at a dose of 1,500 mg/kg body-weight/day. A dose of YKSCH was determined by following the formula for the FDA's human equivalent dose guidance (20). Human equivalent volume (mg/kg) = animal dose (mg/kg) × [Weight_animal_ (kg)/Weight_human_ (kg)]^(1−0.67)^. The human weight was calculated as 60 kg, and the weight of mice was calculated as 0.03 kg. The YKSCH dosage prescribed clinically is 7,500 mg/day. According to the above formula, the dosage in mice is calculated as 1535.5 mg/kg body-weight/day.

**Table 1:** Medical herb composition of YKSCH.

**Common name**	**Weight (g)**
Pinellia tuber	5
Atractylodes rhizome	4
Poria sclerotium	4
Cnidium rhizome	3
Citrus unshiu peel	3
Japanese Angelica root	3
Bupleurum root	2
Glycyrrhiza	1.5
Uncaria hook	3

### High-Performance Liquid Chromatography Analysis of YKSCH

YKSCH extract was mixed and shaken with 50% MeOH, and the supernatant was subjected to high-performance liquid chromatography (HPLC) analysis. The HPLC profile of YKSCH was obtained using a Shimazu LC-20AD liquid chromatography equipped with a SPD-M30A detector with a scanning range of 245 nm and a reversed-phase column (YMC-pack ProC18, 2.0 mm i.d. × 150 mm, 12 nm, column temperature: 20°C). The column was equipped with solvent A (0.1% formic acid in acetonitrile) and solvent B (0.1% formic solution), and the ratio of solvent A was increased from 5% to 70% over 90 min, and remained at 70% over 10 min, with a flow rate of 0.2 mL/min.

### Pentobarbital-Induced Sleeping Model

**Figure 1:**
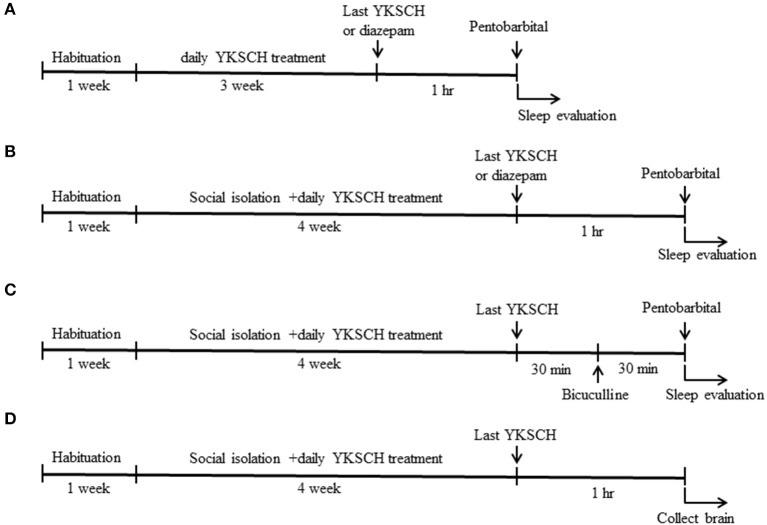
Time schedule of the experiment. Six-week-old male ddy mice were used in this study. Mice were pretreated with 1,500 mg/kg YKSCH for 3 weeks in a group-housed mouse study **(A)** or for 4 weeks in a social isolation stress study **(B)**. One hour after the last treatment, mice were treated with 60 mg/kg pentobarbital. For the GABA receptor antagonist study, mice were treated with 3 mg/kg bicuculline 30 min after the last YKSCH treatment in socially isolated mice **(C)**. For the ELISA and western blot analysis, socially isolated mice were treated with YKSCH for 4 weeks, and mice were sacrificed 1 h after the last YKSCH treatment **(D)**.

Male ddy mice were divided into three groups so that their average body weight is almost the same in each group: control group, YKSCH (1,500 mg/kg)-treated group, and diazepam-treated group. Mice were administered distilled water or 1,500 mg/kg YKSCH for 3 weeks. One hour after the last injection, mice were treated with pentobarbital (60 mg/kg, intraperitoneally (i.p.); Wako industry, Osaka, Japan). As a positive control, diazepam (1 mg/kg, i.p.) was administered once 1 h before pentobarbital injection (Figure 1A). The mice were considered asleep if they stayed immobile and lost their righting reflex when positioned on their back. The time interval between injection of pentobarbital and the start of sleep was noted as sleep latency. Sleep latency and total sleeping time were determined for each mouse. The mice were considered awake if they returned to the upright position. In the test, an equal number of animals from each group were examined once, and the test was performed two times. We checked whether the pentobarbital solution leaked from the mice or not, and the mice the pentobarbital solution leaked from were excluded from analysis. The observer was blind to the treatment.

### Social Isolation Stress-Induced Insomnia Model

Mice were divided into 4 groups so that their average body weight is almost the same in each group: control group (*n* = 8), social isolation stress group (*n* = 8), social isolation stress + YKSCH (1,500 mg/kg)-treated group (*n* = 8), and social isolation stress + diazepam-treated group (*n* = 8). YKSCH was orally administered once daily for 4 weeks. One hour after the last injection, mice were treated with pentobarbital (60 mg/kg, i.p.). Sleep latency and total sleeping time were determined for each mouse. As a positive control, diazepam was administered 1 h before pentobarbital injection (Figure 1B).

To examine whether the effect of YKSCH was mediated by the GABAergic neurotransmitter system, we performed an antagonist study in this model. After 30 min of administration of YKSCH, mice were treated once with 3 mg/kg of bicuculline (Sigma Aldrich, MO, USA), a GABA_A_ receptor antagonist. Then, 30 min later, mice were injected with 60 mg/kg of pentobarbital (Figure 1C). In the test, an equal number of animals from each group was examined once, and the test was performed twice. We checked whether the pentobarbital solution leaked from the mice or not, and the mice the pentobarbital solution leaked from were excluded from the analysis. The observer was blind to the treatment.

### Locomotor Activity Test

For the measurement of locomotor activity, a given mouse was placed in a cage [plastic cage (175 × 245 × 125 mm), with wood-chips, food, and water], and locomotion was measured every 30 min for 2 days using a digital counter with an infrared sensor (Muromachi kikai Co., Ltd, Tokyo, Japan). Animals were placed in the cages at 8:00 a.m. for a 48 h period. To evaluate the effect on locomotor activity, we used the last 24 h of data for analysis.

### Measurement of Allopregnanolone Content

To clarify the mechanisms of YKSCH, mice were randomly divided into 3 groups: control group (*n* = 5), social isolation stress group (*n* = 5), social isolation stress + YKSCH (1,500 mg/kg)-treated group (*n* = 5). Mice were subjected to social isolation stress for 4 weeks with or without YKSCH treatment. One hour after the last treatment with YKSCH, mice were decapitated, and brains quickly removed from the skull, briefly washed in ice-cold saline, laid on a cooled (4°C) metal plate, and both the OB and PFC were rapidly dissected out (Figure 1D). The tissues (10 mg) were added to 100 μL ethanol and shaken vigorously for 30 min, and then centrifuged at 5,000 rpm for 15 min. Supernatants were removed and transferred to new clean tubes and evaporated to dryness under nitrogen. Then, 100 μL ethanol and 400 μL assay buffer were added to dissolve the sample and vortexed well. The extracted samples were used for immunoassays. The ALLO content in the OB and PFC was assayed using ELISA (Arbor Assays, MI, USA).

### Western Blot Analysis

Mice OB and PFC were homogenized in 10 mL/g RIPA buffer (WAKO) supplemented with protease inhibitor cocktail (Sigma Aldrich) and phosphate inhibitor cocktails 2 and 3 (Sigma Aldrich). Lysates were centrifuged at 15,000 g for 20 min at 4°C. An aliquot of 10 μg of protein was subjected to 10–20% sodium dodecyl sulfate–polyacrylamide gel electrophoresis, with the separated protein being transferred onto a polyvinylidene difluoride membrane (Immobilon-P; Millipore, MA, USA). For immunoblotting, the following primary antibodies were used: rabbit anti-SRD5A1 polyclonal antibody (1:1,000; ABclonal technology, MA, USA) and mouse anti-β-actin monoclonal antibody (1:1,000; Cell Signaling Technology (CST), MA, USA). Secondary antibodies were as follows: horseradish peroxidase (HRP)-conjugated goat anti-rabbit IgG (1:5,000; CST) or HRP-conjugated goat anti-mouse IgG (1:5,000; CST). Immunoreactive bands were visualized using LAS-2,000. Band intensity was measured using Image J (NIH, MD, USA).

### Statistical Analysis

All statistical analyses were performed with EZR (Saitama Medical Center, Jichi Medical University, Saitama, Japan), which is a graphical user interface for R (The R Foundation for Statistical Computing, Vienna, Austria). More precisely, it is a modified version of R commander designed to add statistical functions frequently used in biostatistics (21). All data are expressed as mean ± standard error of the mean (SEM). Statistical comparisons were performed using a one-way analysis of variance (ANOVA) followed by Peritz's *F*-test. Differences with *p* < 0.05 were considered statistically significant.

## Results

### HPLC Analysis of YKSCH

**Figure 2:**
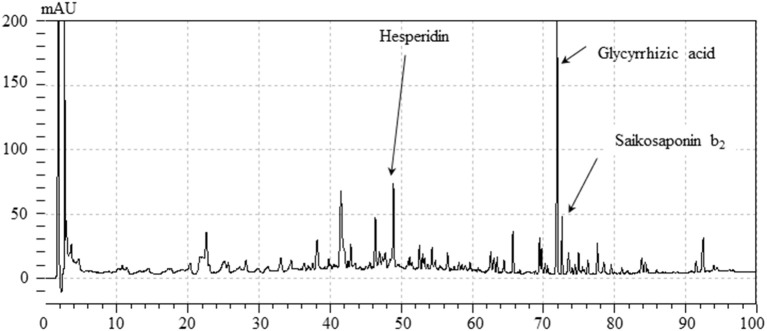
HPLC profile of YKSCH.Each chemical marker (Saikosaponin b_2_, hesperidin, glycyrrhizic acid) in the HPLC profile was identified by comparison with retention times and UV spectra (245 nm) of their reference standards.

Figure 2 shows a HPLC profile of YKSCH along with a chemical analysis at 254 nm wavelength. Chemical markers, such as saikosaponin b_2_, hesperidin and glycyrrhizic acid, were used for quality control.

### Effect of YKSCH on Pentobarbital-Induced Sleep Behavior in Group-Housed or Socially Isolated Mice

**Figure 3:**
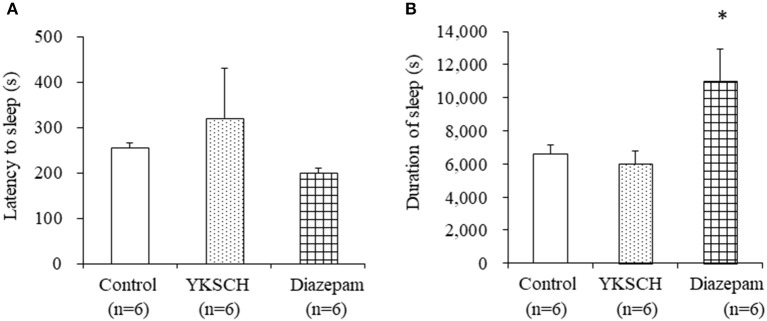
Effect of 3-week treatment with YKSCH on pentobarbital-induced sleeping behavior in group housed mice. One hour after the last injection, mice were treated with pentobarbital. Sleep latency **(A)** and duration **(B)** were measured. Diazepam was administered once, 1 h before pentobarbital injection. Data are expressed as the mean ± SEM (*n* = 6). *p < 0.05 vs. the control group, Peritz's *F*-test.

We first evaluated the effect of YKSCH on a pentobarbital-induced sleep model that is widely used to evaluate sedative drugs. Mice were treated with 1,500 mg/kg YKSCH for 3 weeks before pentobarbital injection. As a result, both the latency and duration of sleep were not affected by YKSCH treatment [sleep latency; *F*_(2, 15)_ = 0.913, *p* = 0.422, sleep duration; *F*_(2, 15)_ = 4.959, *p* = 0.022]. Conversely, treatment with diazepam prolonged the duration of sleep significantly (Figure 3).

**Figure 4:**
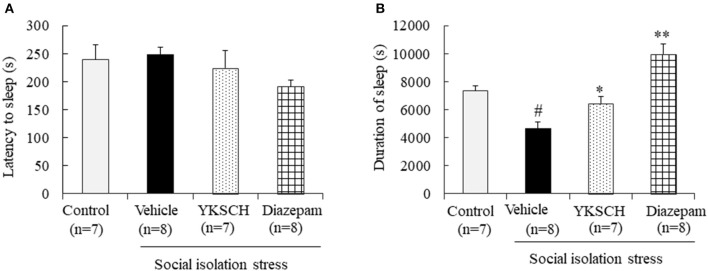
Effect of YKSCH on pentobarbital-induced sleeping behavior in socially isolated mice. Mice were housed individually and treated with YKSCH or vehicle for 4 weeks. One hour after the last injection, mice were treated with pentobarbital. Sleep latency **(A)** and duration **(B)** were measured. Diazepam was treated once 1 h before pentobarbital injection. Data are expressed as the mean ± SEM (*n* = 7–8). ^#^*p* < 0.05 vs. the control group; *p < 0.05, **p < 0.01 vs. the vehicle-treated group, Peritz's *F*-test.

Next, we evaluated the effect of YKSCH using a social isolation-induced insomnia model. In this experiment, social isolation stress decreased the sleep duration but had no effect on sleep latency. Treatment with YKSCH significantly improved the decrease in sleep duration [sleep latency; *F*_(3, 26)_ = 1.524, *p* = 0.232, sleep duration; *F*_(3, 26)_ = 17.73, *p* < 0.001]. Conversely, treatment with diazepam also affected sleep duration significantly (Figure 4). However, YKSCH has no effect on 24 h locomotor activity in the social isolation model (Supplemental Figure 1).

**Figure 5:**
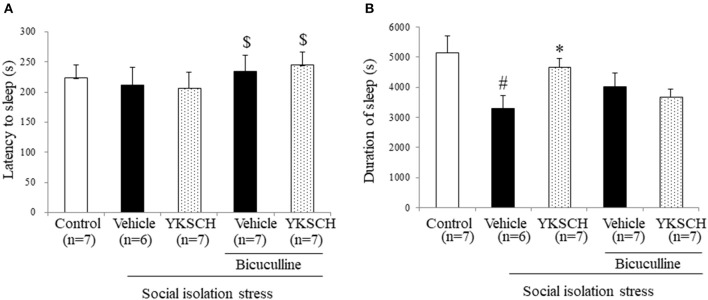
Effect of YKSCH was mediated by GABAergic neuron system in socially isolated mice. Mice were housed individually and treated with YKSCH or vehicle for 4 weeks. Thirty minutes after the last injection, mice were treated with 3 mg/kg bicuculline (i.p.). Mice were treated with 60 mg/kg pentobarbital 30 min after bicuculline injection. The sleep latency **(A)** and duration **(B)** were measured. Diazepam was administered once 1 h before pentobarbital injection. Data are expressed as the mean ± SEM (*n* = 6–7). ^#^*p* < 0.05 vs. the control group; *p < 0.05, vs. the vehicle-treated group; ^$^*p* < 0.05, vs. the YKSCH-treated group, Peritz's *F*-test.

According to previous reports, social isolation stress disrupts many kinds of brain networks, including the GABAergic neuron system. In this study, we investigated whether the effect of YKSCH is related to the GABAergic neuron system by using bicuculline, a GABA receptor antagonist. Treatment with bicuculline did not change either sleep latency or sleep duration in socially isolated mice. On the other hand, prolonged sleep duration with YKSCH treatment was blocked by bicuculline treatment (Figure 5) [sleep latency; *F*_(4, 29)_ = 3.701, *p* = 0.0149, sleep duration; *F*_(4, 29)_ = 4.378, *p* = 0.0068].

### Effect of YKSCH on ALLO and SRD5A1 Expression in the OB and PFC

**Figure 6:**
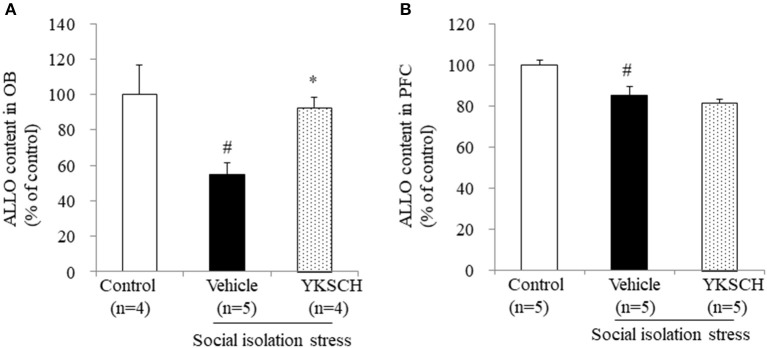
Effect of YKSCH on allopregnanolone content in the olfactory bulb and prefrontal cortex. Allopregnanolone content in the OB **(A)** and PFC **(B)**. Data are expressed as the mean ± SEM (*n* = 4–5). ^#^*p* < 0.05 vs. the control group; *p < 0.05 vs. the vehicle-treated group, Peritz's *F*-test.

A previous report have showed that social isolation stress-induced behavioral abnormalities are related to a reduction in ALLO contents in the OB and PFC (22). In this context, we evaluated the ALLO content in both brain regions. We showed that 4 weeks of isolation-induced stress reduced the ALLO content in both brain regions. Importantly, YKSCH treatment inhibited the decrease in ALLO content in the OB, but not in the PFC (Figure 6) [OB; *F*_(2, 10)_ = 7.083, *p* = 0.0121, PFC; *F*_(2, 12)_ = 11.93, *p* = 0.0014].

**Figure 7:**
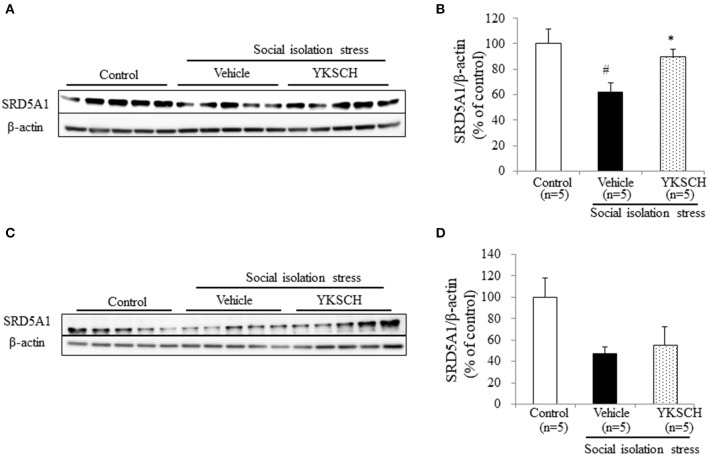
Effect of YKSCH on SRD5A1 expression in the olfactory bulb and prefrontal cortex. **(A,B)** Western blot analyses of SRD5A1 expression in the OB. **(A)** Representative images of immunoblots obtained using antibodies against SRD5A1 and β-actin. **(B)** Quantitative analyses of OB SRD5A1 in socially isolated mice. **(C,D)** Western blot analyses of SRD5A1 expression in the PFC. **(C)** Representative images of immunoblots obtained using antibodies against SRD5A1 and β-actin. **(D)** Quantitative analyses of PFC SRD5A1 in socially isolated mice. Data are expressed as mean % of control ± SEM (*n* = 5). ^#^*p* < 0.05 vs. the control group; *p < 0.05 vs. the vehicle-treated group, Peritz's *F*-test.

Brain cells synthesize ALLO from progesterone by two types enzymes, SRD5A1 and 3α-hydroxysteroid oxidoreductase. In socially isolated mice, the expression level of SRD5A1 is reduced in the OB and PFC, but 3α-hydroxysteroid oxidoreductase is unaffected (19). This report indicates that SRD5A1 is responsible for producing ALLO in this model. In this study, we investigated whether YKSCH treatment affected the SRD5A1 expression level in the OB and PFC. As a result, YKSCH treatment improved the reduction of SRD5A1 expression levels in the OB. However, YKSCH treatment had no effect in the PFC (Figure 7) [OB; *F*_(2, 12)_ = 5.503, *p* = 0.0201, PFC; *F*_(2, 12)_ = 2.786 *p* = 0.101].

## Discussion

Here, we showed YKSCH could improve social isolation stress-induced insomnia via GABAergic actions. In addition, YKSCH treatment improved the ALLO content and SRD5A1 expression level in the mice, especially in the OB.

Benzodiazepines are widely used to treat insomnia, however, benzodiazepines often cause side effects such as sleepiness, amnesia, and dizziness. Therefore, treatment with benzodiazepines is not recommended for elderly people in Japan because of the potential for abuse, dependence, and adverse effects. For that reason, there is a need to develop new drugs with a different mechanism of action than benzodiazepines. Diazepam, a member of the benzodiazepine family, is a positive allosteric modulator of the GABA_A_ receptor. Diazepam works by binding to the GABA_A_ receptor subunit directly and enhancing the affinity of GABA for its binding site. In a previous clinical trial, Aizawa et al. demonstrated that YKSCH treatment in healthy subjects extended the total sleeping time significantly, had no influence on rapid eye movement (REM) sleep, tended to increase stage 2 sleep, and decreased stage 3+4 sleep compared with Anchu-san as the control drug (3). Benzodiazepine treatment was also reported to affect the same sleep stage as that observed with YKSCH treatment. Therefore, this report concluded that the effect of YKSCH may have a similar mechanism of action to that of benzodiazepines in terms of non-REM sleep. However, in the present study, we showed that diazepam treatment significantly prolonged sleep duration in both models. On the other hand, YKSCH treatment improved sleep duration in socially isolated mice only (Figures 2, 3). Among the components of YKSCH, Japanese Angelica root extract improves pentobarbital-induced sleep in socially isolated mice without affecting pentobarbital-induced sleep behavior in group-housed mice (23). Here, we did not investigate which component is responsible for the effect of YKSCH on sleep; however, Japanese Angelica root might partly contribute to the effects of YKSCH.

The abnormal behavior induced by social isolation involves functional changes in various brain neurotransmitter systems, including dopaminergic (13), serotonergic (14), and noradrenergic systems (15, 16). Yokukansan, which is the Chinese medicine that excludes both C. unshiu peel and pinellia tuber from YKSCH, also improves sleeping duration but only in socially isolated mice and not in group-housed mice. Furthermore, Yokukansan improved the shortening of sleeping duration via the GABAergic and not the serotonergic neurotransmitter system in a GABA or 5-HT receptor antagonist study (24). In the present study, we also demonstrated that the effect of YKSCH on a social isolation stress model was blocked by bicuculline injection (Figure 4). This result indicates that long-term YKSCH treatment improved GABAergic neuron disruption induced by social isolation stress and that this mechanism may be similar to that of Yokukansan. And this result also indicates that YKSCH and diazepam have similar mechanisms in that both drugs affect the GABAergic neuron system to improvement social isolation-induced insomnia. Some crude drugs contained in YKSCH (Bupleurum root, Japanese angelica root, and Cnidium rhizome) are reported to have compounds that bind to the GABA binding sites of the GABA_A_ receptor in a receptor binding assay (25). According to previous studies, oral administration of GABA prolonged pentobarbital-induced sleep duration in group-housed mice (26). Though Yokukansan includes water-soluble botanic GABA (27), orally administered GABA does not cross the blood-brain barrier efficiently, and YKSCH treatment could not prolong the sleeping time in group-housed mice (Figure 2). Therefore, we hypothesized that YKSCH might potentiate the GABAergic neuron system through an indirect pathway, such as ALLO.

ALLO is a neuroactive steroid derived from progesterone, and acts as positive allosteric modulator of GABA_A_ receptor action. As a positive allosteric modulator of the GABA_A_ receptor, ALLO has been reported to be an anxiolytic, anesthetic, and anticonvulsant agent (28–30). In addition, ALLO treatment has been reported to improve memory impairment and BPSD-like behavior in Alzheimer's disease mouse models (22, 31, 32). Both ALLO and benzodiazepines are positive allosteric modulators of the GABA_A_ receptor and enhance the GABAergic neurotransmitter system, however, there may differ in terms of side effects, such as tolerance development and abuse liability (33). Several reports have shown that social isolation stress-induced behavioral abnormalities are related to the reduction in ALLO contents in the OB and PFC (22). The ALLO content is reported to be about twice higher in the OB than that in PFC (22). Olfactory dysfunction is frequently observed in patients with REM sleep behavior disorders, such as dementia and Parkinson's disease (34, 35). In addition, some reports have shown that olfactory bulbectomy in rats induces a reduction in REM sleep duration and frequency (36). These reports suggest that the OB has an important role in sleep-wakefulness patterns. Biosynthesis of ALLO in the brain is regulated by two specific enzymes. It starts with the conversion of progesterone into 5α-dihydroprogesterone by the SRD5A1 enzyme. Next, 5α-dihydroprogesterone is converted into ALLO by the 3α-HSD enzyme. Social isolation stress does not change the brain's content of progesterone and pregnenolone (29). In socially isolated mice, brain expression of SRD5A1 mRNA and protein was approximately 50% less than in group-housed mice, whereas the expression of 3α-HSD mRNA was unchanged (19). These reports suggest SRD5A1 is the enzyme responsible for producing the brain ALLO in this model. Here, we demonstrated that YKSCH treatment inhibited the decrease in both ALLO content and SRD5A1 expression levels in the OB (Figures 5, 6). On the other hand, Yokukansan administered once just before pentobarbital injection had the ability to improve the reduced sleep duration in socially isolated mice (24). Although YKSCH could have other rapid mechanisms to improve sleep disruption, the present study suggests that the increase in ALLO content and SRD5A1 expression might be involved in the mechanism of YKSCH in part. However, further studies are required to clarify the mechanisms mediating the action of YKSCH.

There are some limitations to this study. The first is in Figures 3, 4. In the group-housed and social isolation stress model animals, mice were treated with YKSCH for different periods. A previous report showed that social isolation stress for 4 weeks not 3 weeks reduced the sleep duration induced by pentobarbital, so we set the experimental condition based on that study in the social isolation stress model (37). While we performed YKSCH treatment for 3 weeks in group-housed mice, it is possible that YKSCH treatment for 4 weeks might have a similar effect to the effect observed in the social isolation stress model. The second is in Figure 5. There was no significant difference between the YKSCH group and bicuculline + YKSCH group with regard to sleep duration. In the GABA receptor antagonist study, we administered bicuculline once just before pentobarbital injection. This might be the reason why bicuculline treatment did not inhibit the effect of YKSCH completely.

In conclusion, this study demonstrates for the first time that YKSCH treatment improves social isolation stress-induced insomnia via the GABAergic nervous system. In addition, YKSCH treatment increased the ALLO content and SRD5Al expression level in the mouse brain. Moreover, these results suggest that YKSCH may be a good candidate drug to treat patients with insomnia.

## Data Availability Statement

The datasets generated for this study are available on request to the corresponding author.

## Ethics Statement

The animal study was reviewed and approved by the Experimental Animal Care Committee of Kracie Pharma, Ltd.

## Author's Note

This is a Japanese language translation/reprint of 不眠モデルマウスに対する抑肝散加陳皮半夏の効果 originally published in phil 漢方 (phil Kampo) 70, 26–27, 2018. Permission was granted by Medical Publisher Inc. (Japan).

## Author Contributions

KM, NF, and RT contributed to the conception and design of the study. KM conducted all experiments, analyzed the data, and wrote the manuscript. FL, KS, and MO conducted part of the experiments with KM. NF and RT revised the manuscript. All authors gave final approval of the version to be published.

### Conflict of Interest

All authors are employees of Kracie Pharma, Ltd.

## Supplementary Material

Supplemental Figure 1 Effect of YKSCH on locomotor activity in socially isolated mice. **(A,B)** Locomotor activity test. **(A)** Number of beam-breaks throughout the 24 h period. **(B)** Number of beam breaks analyzed separately for day and night. Data are expressed as mean number of beam-breaks ± SEM (*n* = 8).
