# Supplementary figures and images for: Yokukansankachimpihange Improves the Social Isolation-Induced Sleep Disruption and Allopregnanolone Reduction in Mice
Source: Front Nutr. 2020 Feb 11;7:8. doi: 10.3389/fnut.2020.00008 (PMC7026005; doi:10.3389/fnut.2020.00008)

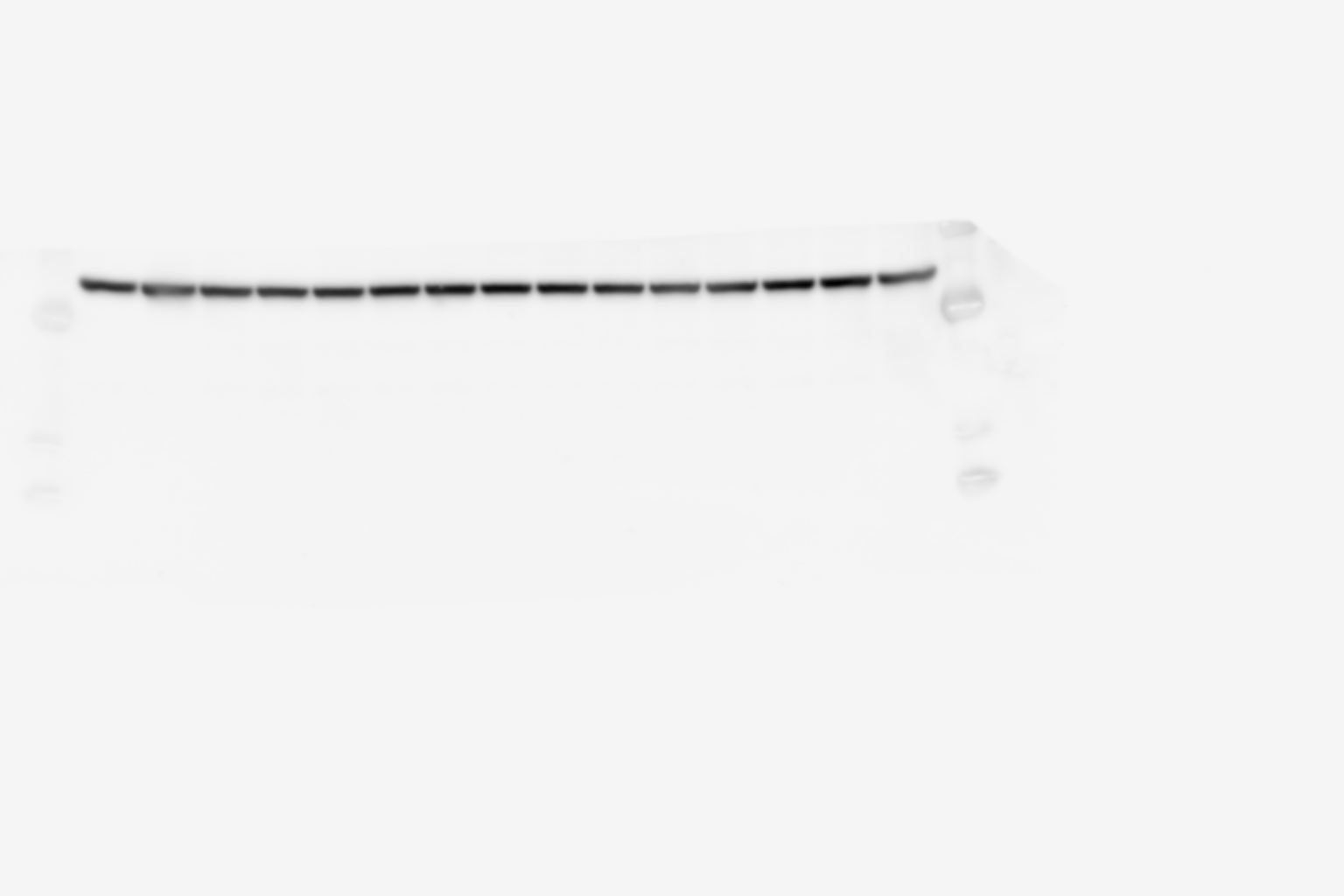

Supplement: Supplemental Figure 1 — Effect of YKSCH on locomotor activity in socially isolated mice. (A,B) Locomotor activity test. (A) Number of beam-breaks throughout the 24 h period. (B) Number of beam breaks analyzed separately for day and night. Data are expressed as mean number of beam-breaks ± SEM (n = 8). [file Image_1.TIF]

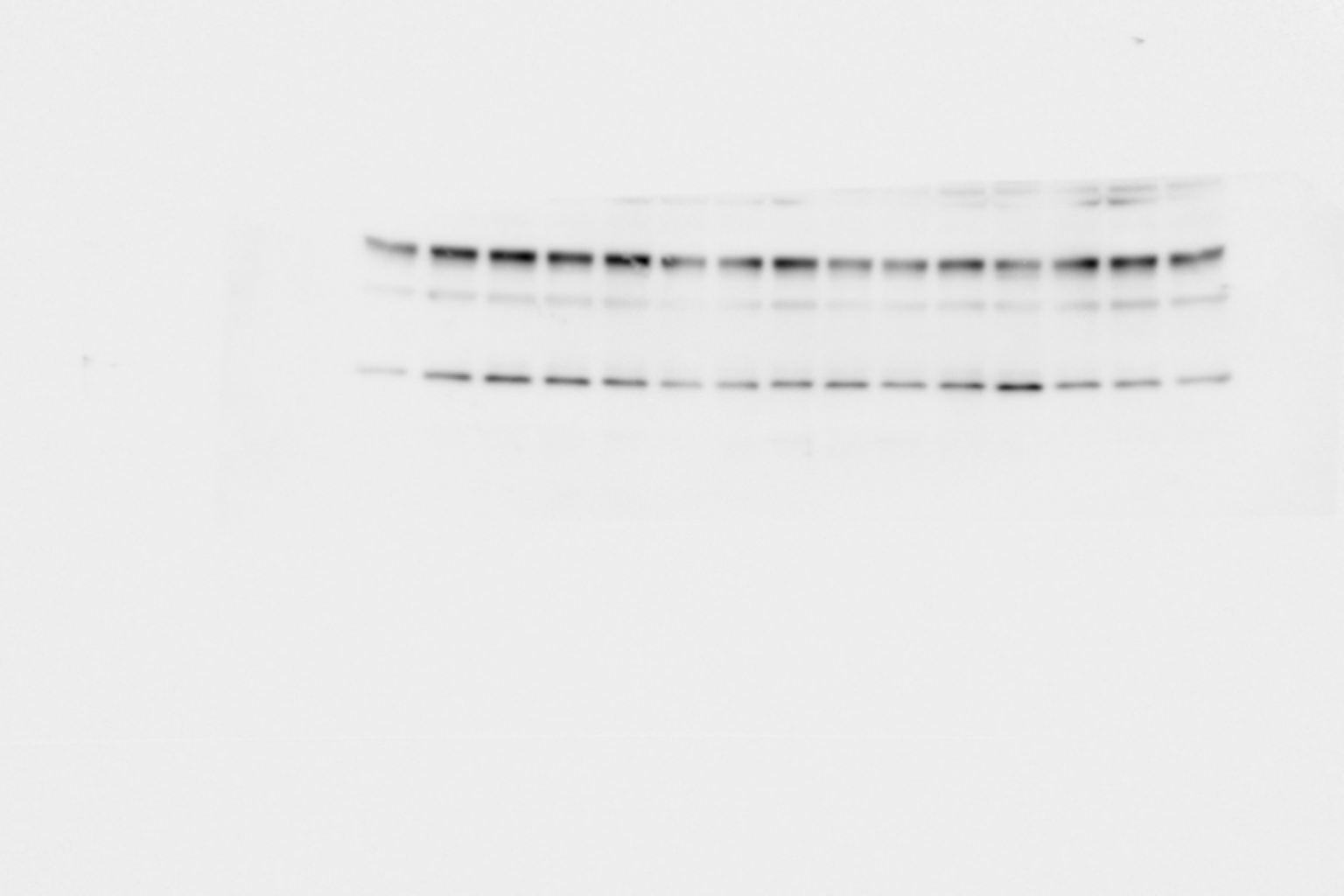

Supplement: Supplementary file 2 [file Image_2.TIF]

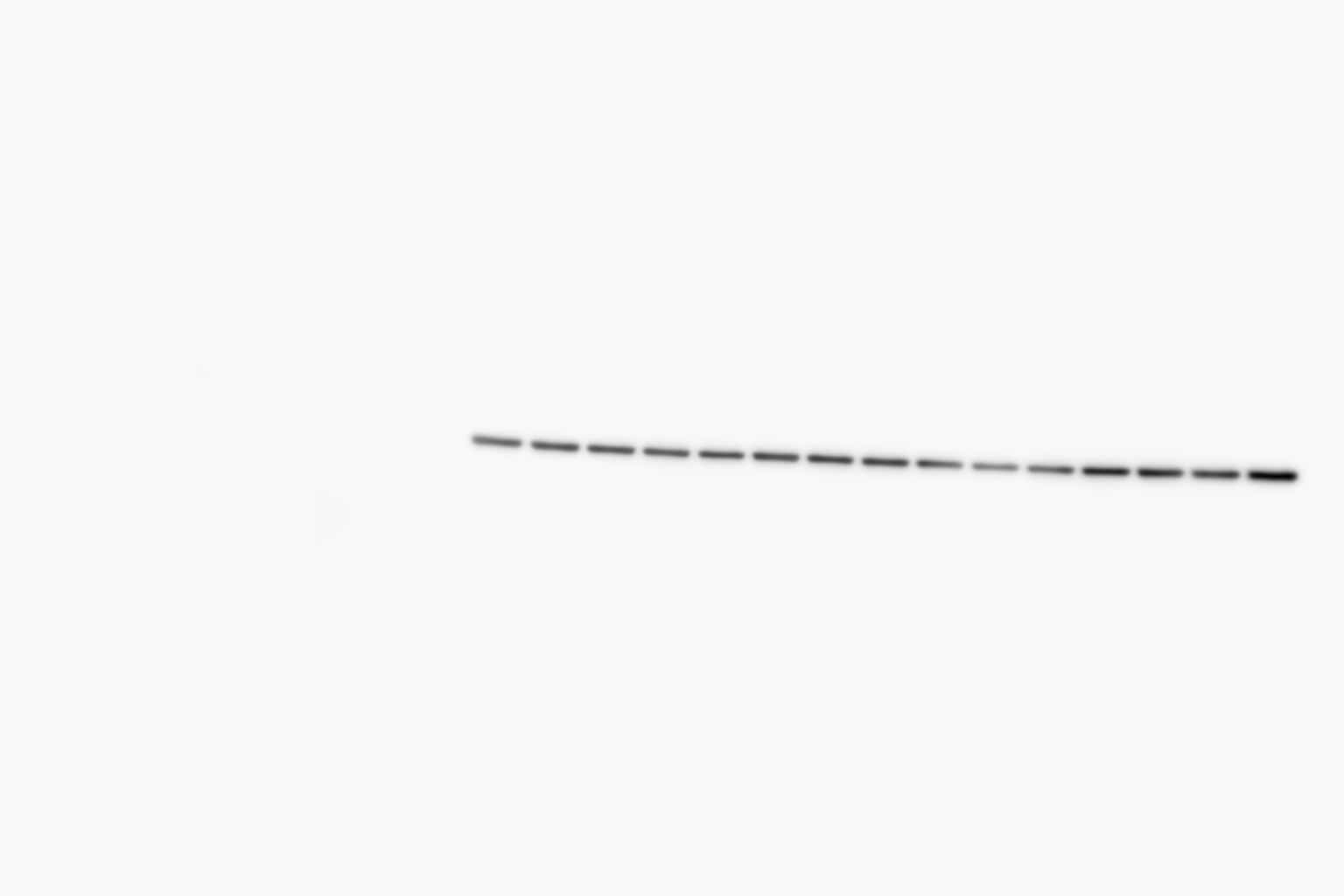

Supplement: Supplementary file 3 [file Image_3.TIF]

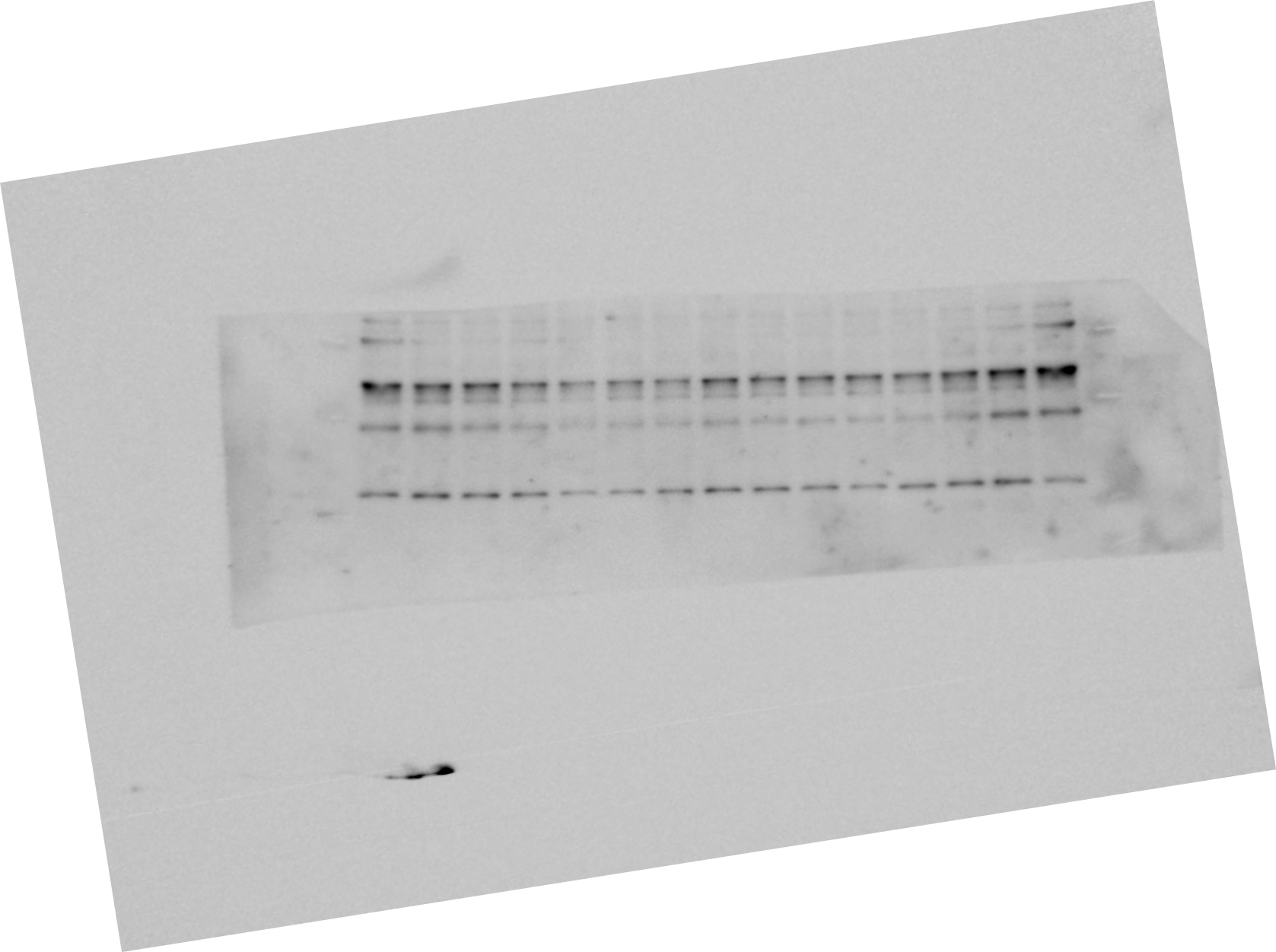

Supplement: Supplementary file 4 [file Image_4.TIF]
